# The role of emergency preparedness exercises in the response to a mass casualty terrorist incident: A mixed methods study

**DOI:** 10.1016/j.ijdrr.2020.101503

**Published:** 2020-06

**Authors:** Elena A. Skryabina, Naomi Betts, Gabriel Reedy, Paul Riley, Richard Amlôt

**Affiliations:** a Behavioural Science, Emergency Response Department Science & Technology, Public Health England, Porton Down, Salisbury, Wiltshire, SP4 0JG, UK; b Faculty of Life Science and Medicine, King's College London, Waterloo Bridge Wing 5.14, London, SE1 8WA, UK; c Principal Expert Emergency Preparedness and Response, European Centre for Disease Prevention and Control (ECDC), Gustav III:s boulevard 40, 169 73 Solna, Sweden; d Emergency Response Department Science & Technology, Public Health England, Porton Down, Salisbury, Wiltshire, SP4 0JG, UK

**Keywords:** Emergency medical services, Emergency preparedness, Emergency preparedness exercises, Manchester Arena bombing, HEPE, Disaster training

## Abstract

Simulation exercises are an important part of emergency preparedness activities for the healthcare community but evidence of their impact on the response to real major incidents is limited. This project studied the impact of health emergency preparedness exercises (HEPEs) on the response to a mass casualty terrorist incident. The mixed methods study design was adopted comprising an on-line survey and follow up individual interviews. Participants were healthcare staff who took part in responses to three major terrorist incidents in the UK in 2017. Descriptive statistics and analysis of variance were undertaken with quantitative data. Content and thematic analysis methods were used for qualitative data analysis.

The online survey generated 86 responses; 79 (92%) were from the responders to the Manchester Arena bombing. Twenty-one survey respondents shared their experiences in in-depth interviews. Healthcare staff who took part in HEPEs felt better prepared to respond than those who did not attend an exercise. The most commonly reported benefits from HEPEs were awareness of major incident plans and having the opportunity to practice responding to a similar scenario in the recent exercise. Specific benefits included: improved coordination of the response through adherence to recently practiced incident plans*;* confidence with response roles; real-time modifications of the response and support provided to staff who did not take part in exercises. Exercise recency was highlighted as an important facilitating factor.

The study provides strong objective evidence that the response to a mass casualty terrorist incident was enhanced by training and service development achieved through HEPEs.

## Abbreviations

CCG: Clinical Commissioning Group
CT: Computerised Tomography
ED: Emergency Department
EPE: Emergency Preparedness Exercise
EPRR: Emergency Preparedness, Resilience and Response
ERD: Emergency Response Department
GM: Greater Manchester
HEPE: Health Emergency Preparedness Exercises
ICC: Incident Coordination Centre
PHE: Public Health England
PHE REGG: Public Health England Research Ethics and Governance Group
NHS: National Health Service
NHS Trust: an individual hospital, community medicine provider or other organisation providing patient care services as part of the National Health Service
TTX: Tabletop Exercise

## 1 Introduction

Multiple acts of terrorism in 2017 tested UK emergency response arrangements and put the health system's preparedness to respond to a mass casualty major incident into focus. The Manchester Arena bombing alone left 22 people dead, 116 people requiring hospital treatment, and many more with psychological and emotional trauma [1,2]. As such, these types of events put significant pressure on the health system during and after an incident, and it is essential that services and personnel are well-equipped and prepared to respond.

Emergency preparedness exercises are an important part of emergency preparedness activities for the healthcare community. NHS England EPRR Core Standards [3] require that hospitals, as a part of their emergency preparedness, resilience and response planning, must conduct at least one communication exercise every six months, a desktop exercise once a year, and a major live exercise and a command post exercise every three years. As a result, health emergency response organisations participate in a variety of health emergency preparedness exercises (HEPEs) that are designed to test and improve individual and organisational capacity to respond in a major incident.

The potential benefits from participation in HEPEs, both for individuals and organisations involved, have been outlined in the literature [4]. Internationally, exercises are reported to help to clarify healthcare staff roles and responsibilities, and to help identify health system challenges [[5], [6], [7]]. However, despite growing evidence of immediate (post-exercise) benefits, there is limited empirical evidence on the impact of these often expensive and complex training activities on the response to real major incidents [4,8]. This is mainly because of the comparative rarity and unpredictability of mass casualty incidents: the contribution of particular training or preparedness activities to the effectiveness of a response is hard to study. A few studies where the impact of exercises on real major incident response have been discussed [[9], [10], [11]] are relatively descriptive and lack methodological rigor with data collection and analysis. Despite these difficulties, disseminating the impacts of exercises can promote continued engagement by health professionals [12] and can sustain interest in exercise programs [13].

Previously planned health emergency preparedness exercises were undertaken shortly before both the Manchester and London attacks in the UK in 2017 [14]. This provided an opportunity to study the impact of those exercises on the subsequent responses. The present study focuses on reporting the impact of two HEPEs, Exercise Elsa and Exercise Socrates, delivered in March 2017, on UK healthcare professionals’ response to the Manchester Arena bombing on the 22 May 2017 (participants involved in London major incidents of 2017 (the Westminster Bridge attack and the London Bridge attack) were also surveyed for this research, but a very low response rate was achieved and as such the study focuses on the response to the Manchester Arena attack). In this report, for the first time to our knowledge, rigorous empirical evidence is provided from a large-scale, mixed methods study, regarding how participation in these exercises contributed to the response to a subsequent large-scale mass casualty terrorist incident. Factors that facilitated the response are discussed, and recommendations for emergency preparedness training are provided based on the findings.

## 2 Background

Emergency preparedness activities consist of many components, including a complex cycle of planning, preparing equipment, training staff, exercising and improvements [15]. Emergency preparedness exercises (EPEs) are often considered the most vital part of the cycle [16,17] because of the potential of dual value: at the individual level, EPEs provide an opportunity for response staff to learn about emergency plans and procedures experientially, through hands-on practice of a simulated response and at the organisational/system level, EPEs help to identify gaps in planning, training and resources [18]. This identification of gaps and limitations has the potential to drive positive changes to improve preparedness [13].

Broadly, emergency preparedness exercises fall into two major groups: discussion-based exercises and operation-based exercises. Discussion-based simulation exercises, also known as tabletop or desktop exercises (and often abbreviated as TTX), allow participants to practise their response roles and emergency plans through facilitated group discussions. In such exercises, participants verbally describe their actions, and engage in facilitated discussions to address challenges posed by the exercise scenario. As such, TTX are considered the least formal type of exercises and they are relatively easy to organise and conduct [13]. Indeed, TTX are recommended to be conducted before more complicated operation-based exercises [[19], [20], [21]]. They are typically conducted in a low-pressure environment to facilitate learning and not under real time conditions; time compression allows consideration of different aspects of preparedness in one exercise. Further, TTX are considered particularly useful when introducing new plans or procedures: they bring multiple agencies together, each providing different functions under the plan, to understand their response abilities and to identify potential gaps and limitations in local response arrangements [18]. The level of discussion and networking that naturally occurs from the mix of participants and agencies involved in a TTX was cited as the most valuable aspect of a TTX [[22], [23], [24]]; indeed, the effectiveness of inter- and intra-organisational relationships is associated with successful performance during a response [25]. Other reported benefits from TTX include improved knowledge of emergency plans, roles and responsibilities and in identifying system-level challenges [5,24,26].

Operation-based exercises involve a functional element and are classified into different types depending on the exercise purpose. Drills are operation-based exercises which are used to test a specific operation or function under a response plan, typically within a single entity and with involvement of operational staff [13]. Functional, or command post exercises (CPX), focus on testing coordination, command, and control between multi-agency coordination centres. A CPX is typically conducted from an emergency operation centre with staff in tactical and strategic command roles [13]. The most complex type of operation-based exercise is a full-scale exercise (FSX), which is conducted to test all major parts of the functions specified in the response plan, and which involves multiple agencies and response staff at all levels [19,25]. Operation-based exercises are normally conducted in real time, under realistic conditions that seek to replicate the environment and pressures typical of an emergency situation. Typically, staff execute their functions under the plan using appropriate equipment [25]. Reported participant benefits from operation-based exercises include improved knowledge of emergency plans and response roles [27], improved perceptions of planning and training adequacy, and improvements in perceptions of teamwork, response network effectiveness, equipment adequacy as well as reduced level of stress associated with a response [15,25]. At an organisational level, operation-based exercises help to identify gaps and limitations in emergency plans, protocols, procedures and training [4]. Operation-based exercises are more time- and resource-intensive to prepare and conduct, compared to TTX. However, this additional commitment of time and resources allows a more thorough testing of plans, tools, procedures, resources, technologies, and command centres under conditions closely matching real events. Practising emergency response under pressure offered by operation-based exercises has highlighted multiple issues and errors that have not been picked up from discussion-based exercises and normal practices [28,29].

Despite multiple benefits reported with both discussion-based and operation-based exercises, there is little empirical evidence of the preparedness exercises’ impact on health response in a major incident [4]. Unlike other emergency responders, such as police and firefighters, healthcare staff are often not engaged routinely in practicing their major incident emergency response skills, and their benefits from exercises may more closely resemble those reported by volunteers, rather than professional responders [25]. There is also little empirical evidence of skills retention and translation to daily practice by healthcare exercise participants, or health systems making improvements to preparedness based on exercises and real events [30,31]. Repeated identification of the same lessons from health emergency preparedness exercises, and from response to real major incidents, indicates that lessons are often not translated into system improvement [31].

However, system and organisational learning from incidents and exercises is crucial to improve preparedness [14]. Models of organisational learning highlight the importance of identification and implementation of learning from exercises [32]. Organisational debriefing plays an important role in identifying lessons from real events and exercises, and further follow-up steps, such as practical recommendations and actions that lead to effective interventions, are necessary [33]. Literature on organisational learning post-incident differentiates single loop learning, when only the specific situation or process is improved, from double-loop learning, which involves learning at a higher level and requires that not only a specific situation is improved, but also that values, assumptions and policies are scrutinized [30,34]. Opportunities for double-loop learning are often missed, due to difficulties in identification of these organisational factors, as well as managerial weaknesses [30,34].

The effectiveness of preparedness activities can be judged by studying the impact of emergency preparedness exercises in the response to a real major incident. However, literature to date is lacking reports of models to assess such an impact; as such, individuals' perceptions are often the main source of information [[9], [10], [11]]. Educational models to evaluate the effectiveness of complex interventions can be offered as one solution. The Kirkpatrick model of training effectiveness [35], often-quoted in health education literature, describes four levels of potential outcome that can be measured from an educational intervention (such as an emergency preparedness exercise). These are: 1. Reactions (how satisfied are participants with the intervention); 2. Learning (what knowledge or skills have been learned from an exercise); 3. Behaviour (what changes in behaviour have occurred as a result of exercise participation) and 4. Results (what impact does an exercise have on the outputs of the system). This model can be used as a framework to evaluate emergency preparedness exercises’ effectiveness, as well as to study the impact of exercises in the response to a real incident.

### 2.1 Exercises

**Exercise Elsa** was a one-day discussion-based tabletop exercise (TTX) that took place on 22 March 2017 in Manchester, UK. The aim was to use a new mass casualty distribution plan while practising the health and social care response to a major incident that involved a significant number of casualties with traumatic injuries, including burns and paediatric casualties. The exercise scenario generated around 400 simulated casualties from two simultaneous incidents, which required a regional multi-agency response. Exercise Elsa was attended by 108 healthcare responders, mainly tactical and strategic commanders, representing various health organisations in the region. The exercise provided an opportunity for participants to familiarise themselves with a new mass casualty distribution plan and to investigate compliance.

**Exercise Socrates** was a one-day operation-based exercise that took place on 29 March 2017 in Manchester, UK. The exercise aimed to test the trauma network response to a mass casualty incident involving traumatic injuries in the Greater Manchester Health System, in collaboration with partner agencies, to support the further development of the proposed Greater Manchester mass casualty distribution plan. The real-time exercise was based on a scenario of a simultaneous suicide bombing and marauding terrorist firearm attack (MTFA) at Manchester airport, resulting in a significant number (187) of adult and pediatric casualties. The exercise used the Emergo Train System® [36] format and was a hybrid of two types of operation-based exercises: a functional/command post exercise (CPX), where coordination of casualties distribution from the scene of the incident within a trauma network was practiced using a new mass casualty distribution plan, and a response drill, where casualties management at the scene and within a hospital pathway was practiced. Ambulance services used a mass casualty distribution plan to distribute patients from the scene to the most appropriate hospitals, using pre-defined hospital capacities, and patient information was sent in real time to the hospitals to practice triage and care planning.

### 2.2 Manchester Arena bombing

**The Manchester Arena bombing** took place on the 22 May 2017 at 22:31, when a suicide bomber detonated an improvised explosive device in the foyer of the Manchester Arena as a 14,000-person crowd was leaving an event. Many in the crowd were teenagers and children accompanied by their parents. The attack claimed the lives of 22 people. A further 116 people required medical treatment, mostly for ballistic injuries, and many more were left with psychological and emotional trauma [1,2].

## 3 Method

A convergent parallel mixed methods study [37] was conducted, which was designed to explore the experiences of healthcare staff who had responded to a mass casualty major incident; in particular, the focus of the study was to identify the specific components of exercises and training that had helped them to respond to the incident.

The study included an anonymous on-line survey distributed to those who responded to three mass casualty incidents in the UK in 2017 (the Westminster Bridge attack [38], the Manchester Arena bombing [39] and the London Bridge attack [40]); follow-up semi-structured interviews were conducted with some survey participants. The in-depth qualitative data from the interviews was used to complement and explain the survey results and provided more insight into participants’ thinking about HEPEs and their impact on real incident response.

Since there are no established models for evaluating the impact of preparedness exercises on a real response, the convergent mixed methods design [37] was chosen. The intention of the design is to capitalise on the benefits of a larger sample size allowed by the survey, with the added depth and breadth of exploration allowed by interviews [41]. The mixed methods study design, through a combination of quantitative and qualitative data, allows for more exploratory analysis of the phenomenon to be undertaken [42], and contributes additional confidence and accuracy of data interpretation [43].

Ethical approval for the study was provided by Public Health England Research Ethics and Governance Group (PHE REGG R&D298). Individual informed consent for the survey component was completed on-line by participants who completed the survey. A further informed consent process was completed with all interview participants, and signed consent was obtained before the interview was initiated.

### 3.1 Data collection and analysis

#### 3.1.1 Quantitative data

An anonymous on-line survey of responders to the Westminster Bridge attack, the Manchester Arena bombing and the London Bridge attack was conducted between 10^th^ August 2017 and 11^th^ December 2017. Healthcare organisations who took part in the response to these incidents (NHS Ambulance Service, NHS Trusts, Clinical Commissioning Group (CCG), NHS England, Public Health England, NHS Improvement and NHS mental health trusts) were informed of the study and asked to share a link to the on-line survey with their staff. Healthcare staff who responded to any of those 2017 major incidents were eligible to take part in the study. The survey content was designed in consultation with emergency preparedness experts, including emergency preparedness specialists, military personnel, clinicians and members of public and guided by the evidence provided in the research literature (Supplementary material File 1). Primary outcome measures were participants' perceptions of training adequacy, equipment adequacy, clarity of roles and perceptions of their own and their organisation's performance in the response; these are the factors that were previously reported as improved through exercise participation [15,25].

Secondary outcome measures included data for groups' knowledge and activities related to their major incident plan. Improvements in knowledge and understanding of plans were reported from exercise participation [26,27]. Assuming that the considered exercises were successful, it was hypothesised that differences between exercise and non-exercise participants’ perceptions of the above factors will be observed. Between group analysis of variance via one-way ANOVA was conducted to compare perceptions between two groups of health responders (those who took part in either of the recent HEPEs and those who did not). Descriptive statistics were used to check for any differences between exercise and non-exercise groups. IBM SPSS Statistics for Windows, Version 24.0 (Armonk, NY: IBM Corp) was used for the statistical analysis.

#### 3.1.2 Qualitative data

The survey included open-ended questions to allow participants to share their views of the effectiveness of their emergency preparedness training in the response, the exercises’ impact on the response, and to suggest further training to help them in their roles (Supplementary material File 1). Qualitative data obtained from the survey were subjected to summative content analysis [44,45] to identify prevalent factors that contributed to the response. The factors were further analysed to identify emerging themes.

An invitation to take part in follow-up interviews was offered at the end of the on-line survey, and 59 respondents expressed their interest in taking part. Individual interviews were conducted by telephone between October and December 2017, with 21 responders to the Manchester Arena bombing. The average length of interviews was approximately 48 min (range: 27–69 min). The interviews pursued an exploratory aim and adopted a phenomenological strategy [42] to explore participants' experiences openly without any prior assumptions or biases. The semi-structured topical interview schedule was developed in consultation with the project advisory group (comprised of clinicians, emergency preparedness experts, and members of public) and included questions to understand participants' reactions, learning, behaviours and results, as per Kirkpatrick's model. (Supplementary material File 2 (exercise participants) and File 3 (non-exercise participants)).

Participants were asked to share their views of the response and discuss challenges they faced in their roles. A specific focus was to explore their emergency preparedness training and the role of exercises in the response. Participants also discussed skills and knowledge necessary for an effective response to a major incident in their roles and discussed further training to improve their preparedness. Interviews were mainly conducted by the first author (ES), digitally recorded and transcribed verbatim. The transcribed data were then subjected to a thematic analysis using the following approach: 1) becoming familiar with the data; 2) generating initial codes; 3) searching for themes; 4) reviewing themes and 5) defining and naming themes [46]. Coding was undertaken by using NVIVO 11 software (QSR International). Coding reliability and validity was checked by asking another researcher (PR) to independently apply generated codes to two randomly selected transcripts using NVIVO 11. The coding agreement achieved for both transcripts was in the range of 88.3%–99.6%, indicating good agreement and consistency.

In line with a convergent mixed methods design, the analysis of the data from the two strands (quantitative and qualitative) was done independently, and the results were then merged [41]. This allows the two types of data to contribute to a more complete understanding of the phenomenon.

## 4 Results

### 4.1 Quantitative data

#### 4.1.1 Responders’ characteristics

In total, 86 responses were collected from the on-line survey: 79 (92%) from responders to the Manchester Arena bombing, four (5%) from responders to the London Bridge attack and three (3%) from responders to the Westminster Bridge attack. Survey participants were predominantly White British (68, 79%), with male and female gender identification being equally represented. Participants were predominantly aged between 50 and 59 years (40%), with 85% having either graduate or postgraduate education. Most survey participants were from acute NHS trusts (64, 74%), in clinical roles (44, 51%) and the majority (60, 70%) had performed an operational role in the response. Out of 86 responders, 36 (42%) took part in one of the exercises (five participants took part in Exercise Elsa only, 20 in Exercise Socrates, and eight in both; three took part in Exercise Watling Street (London response group)). There were no significant differences between exercise and non-exercise groups with regards to demographic and role-related characteristics (see Table 1).

**Table 1:** Baseline characteristics of responders, separately for two groups: Exercise group (Ex) and Non-exercise group (Non-Ex). Nb. differences in major incident involvement and years of experience were assessed using independent samples t-tests.

	Ex (n = 36)	Non-Ex (n = 50)	*Chi-square*	*df*	*p*
**Participating organisations**					
CCG	1	2			
Acute Trust	27	37			
Ambulance Service	4	1			
NHS England	4	3			
PHE	0	5			
NHS Improvement	0	1			
Mental Health Trust	0	1			
*Total*	36 (42%)	50 (58%)	8.793	6	0.186
**Emergency response role in the incident**					
Operational (Bronze)	22	38			
Tactical (Silver)	8	6			
Strategic (Gold)	0	4			
Support	3	0			
Strategic + tactical	1	0			
Operational + tactical	2	2			
*Total*	36	50	9.731	5	0.083
**Day role**					
EPRR	4	3			
Clinical	16	28			
Managerial	9	11			
Scientific	1	1			
Support	5	3			
Communication	1	1			
*Total*	36	47	3.568	5	0.613
**Major incident response involvement,** times (Mean, SD)	3.4(3.9)	2.6 (3.4)			0.320
**Experience,** years (Mean, SD)	8.6 (9.2)	9.4 (8.2)			0.691
**Gender**					
Female	15	24			
Male	16	23			
Prefer not to say		1			
*Total*	31	48	0.708	2	0.702
**Age**					
18–29	3	3			
30–39	7	5			
40–49	5	18			
50–59	15	19			
60 or over	1	3			
Prefer not to say	2	4			
Total	35	50	7.402	5	0.192
**Education**					
Doctorate degree (e.g. PhD, MBBS)	8	17			
Masters/post graduate degree	10	17			
Undergraduate degree	10	11			
A-level or equivalent	2	0			
GCSE or equivalent	0	2			
Prefer not to say	1	1			
Missing	5	2			
*Total*	36	50	8.330	6	0.215
**Ethnicity**					
British White	28	40			
Irish	1	0			
Any other white background	1	4			
White and Black African	1	0			
White and Asian	0	1			
Indian	0	1			
*Total*	31	46	5.883	6	0.436

#### 4.1.2 Responders’ perceptions

When participants' various perceptions of their own and their organisation's performance in the response were compared, the only difference between the groups emerged in the perceptions of training: health staff who took part in the exercises reported that they had felt significantly better prepared to respond to this major incident when compared to the non-exercise group (F_1,84_ = 10.82; *p* = 0.001; see Table 2).

**Table 2:** Perceptions of effectiveness (primary outcome measures) for Exercise (N = 36) and Non-exercise (N = 50) groups.

Perceptions of the effectiveness of	Group	Mean (SD)	F_1.84_	*p-*value
Personal response	Exercise Non-exercise	4.06 (0.72) 3.94 (0.87)	0.429	0.514
Clarity of the main role (Yes, %)	Exercise Non-exercise	33 (92%) 45 (90%)		0.793^#^
Clarity of team role (Yes, %)	Exercise Non-exercise	32 (89%) 46 (92%)		0.493^##^
Training (personal)	Exercise Non-exercise	3.61 (0.55) 3.06 (0.89)	10.82	0.001**
Training (team)	Exercise Non-exercise	3.36 (0.64) 3.16 (0.84)	1.45	0.232
Training (organisation)	Exercise Non-exercise	3.57 (0.66) 3.44 (0.73)	0.72	0.398
Training (team/others)	Exercise Non-exercise	3.42 (0.77) 3.16 (0.82)	2.17	0.145
Training (organisation/others)	Exercise Non-exercise	3.40 (0.70) 3.10 (0.91)	2.70	0.104
Organisational C3(command, control, coordination)	Exercise Non-exercise	4.33 (0.86) 4.30 (0.84)	0.032	0.858
Organisational Internal Communication	Exercise Non-exercise	3.86 (0.96) 4.00 (0.88)	0.48	0.489
Organisational External Communication	Exercise Non-exercise	3.42 (1.20) 3.64 (1.26)	0.68	0.411
Dealing with public queries	Exercise Non-exercise	3.17 (1.38) 3.40 (1.47)	0.55	0.459
Equipment allocation	Exercise Non-exercise	3.81 (1.33) 3.81 (1.25)	0.00	0.989
Equipment utilisation	Exercise Non-exercise	3.90 (1.27) 3.81 (1.28)	0.103	0.749
Equipment effectiveness	Exercise Non-exercise	3.90 (1.30) 3.78 (1.28)	0.162	0.688
Resources availability (my role)	Exercise Non-exercise	3.53 (0.61) 3.36 (0.75)	1.222	0.272
Resources availability (team)	Exercise Non-exercise	3.42 (0.65) 3.27 (0.76)	0.933	0.337
Resources availability (organisation)	Exercise Non-exercise	3.25 (0.57) 3.43 (0.54)	1.923	0.170

One-way Anova, significance at **p < 0.005; ^#^Pearson Chi-square equal 0.69, df = 1, *p* = 0.793; ^##^ Pearson Chi-square equal 1.414, df = 2, *p* = 0.493.

There were a few differences between the exercise and non-exercise groups with regards to their perceptions of the role of major incident (MI) plans. Significantly more exercise participants had practiced their MI plan over the past 3 years, reported being able to identify limitations in the plan following the response, and also being able to understand how identified limitations could be addressed (see Table 3).

**Table 3:** Perceptions of a Major Incident Plan and post-response activities.

	Exercise group, N = 28	Non-exercise group, N = 45	*p*
Does your organisation have a MI plan? (Yes, %)	28 (100%)	41 (98%)	0.411
Was the plan useful in the response? (Yes, %)	25 (93%)	36 (92%)	0.966
Did you adhere to the plan in the response? (Yes, %)	22 (79%)	30 (79%)	0.791
Have you practiced your plan over the past 3 years? (Yes, %)	25 (89%)	18 (46%)	0.000**
Post event involvement			
Have you taken part in a debrief to identify lessons from the response? (Yes, %)	24 (86%)	35 (78%)	0.402
Have any limitations in plans been identified from the response? (Yes, %)	24 (86%)	25 (64%)	0.049*
Do you understand how limitations in plans will be addressed (Yes, %)	25 (89%)	28 (62%)	0.012*
Have you taken part in a multi-agency debrief? (Yes, %)	23 (82%)	36 (84%)	0.862

Pearson chi-square is reported; significance: *- *p* ≤ 0.05; ** - *p* < 0.005.

When explicitly asked about the exercise's impact on the response, five (38%) of the Exercise Elsa participants (two of whom attended Exercise Elsa and three both Exercises Elsa and Socrates) and 24 (86%) of the Exercise Socrates participants (19 attended Exercise Socrates only and five attended both exercises) stated explicitly that the exercise made a difference to their ability to respond to the subsequent Manchester Arena bombing.

### 4.2 Qualitative data

Thematic analysis of the data obtained from interviews with 21 responders to the Manchester Arena bombing, and the content analysis of the open-ended survey questions obtained from 86 survey participants, helped to understand how exercises contributed to the response.

#### 4.2.1 Responders’ characteristics

Out of 21 interviews completed with health responders to the Manchester Arena bombing, 12 interviews were completed with those who work as clinicians, including seven medical consultants, a consultant clinical psychologist and three nurses. Five further interviews were completed with EPRR staff, three with hospital management and one with a member of staff in a non-clinical role (see Table 4). All except two participants were from acute NHS trusts (N = 19) and included 11 who identified as female and 10 who identified as male. Most participants were aged between 40 and 59 years (n = 13), six others were 18–39 years, and two were older than 60 years. All except two interviewees were British White (n = 19). Participants had a broad range of work experience, ranging between one and 42 years, with median of four years of experience. Thirteen participants attended at least one of the exercises (eight attended Exercise Socrates, one Exercise Elsa, and four attended both) while eight participants did not attend any of these exercises.

**Table 4:** Interview participants’ daily jobs.

Participants daily jobs	No.	Job Category
Consultant	8	Clinical (12)
Nurse	3	
ED advanced practitioner	1	
Divisions governance lead for surgery	1	Managerial (3)
Head of trust communications	1	
Theatre manager	1	
Emergency Planning Officer (EPO)	5	EPRR (5)
Blood transfusion services	1	Non-clinical (1)

#### 4.2.2 Responders’ perceptions

The most frequently occurring themes from the data related to the exercises’ impact on the response are summarised in the sections below and are supported by selected quotes. The themes are discussed in the order of their prevalence. Numbered illustrative quotes from interview participants correspond to their unique survey participant number. An overview of the major themes is provided in Table 5.

**Table 5:** Semi structured interviews – emerged major themes and subthemes.

Theme	Subtheme	Number of Sources	Number of References
Opportunity to practise the response	My role Plans Response	11	28
Knowing what to do		10	23
A predermined plan		8	18
Real-time modification of response		7	15
Addressing lessons from exercises		4	11

### 4.3 Opportunity to practise the response

One of the most common themes that emerged from the qualitative data related to the impact of exercises on the response was the opportunity to practice the response. The opportunity to practice responding to a large-scale mass casualty incident offered by both simulation exercises (Elsa and Socrates), but particularly by operation-based Exercise Socrates, was mentioned most often by responders as a factor that contributed to their response in the Manchester Arena bombing. The practice helped participants to develop better understanding of a system-wide response to a major incident and their response roles; it also gave participants experience which many had not had before, and which was considered as a rehearsal of their actions for the real incident they subsequently found themselves responding to:

*…the opportunity for us all to have practiced what we ended up doing in reality after the bomb, and working together, recognising each other’s roles. In a huge hospital such as ours - many of us never see each other, do not know each other - and have no idea what others do. Socrates was really useful in many of us appreciating each other’s roles*. [33]

*I think had I not have had those exercise training services I don't believe I'd have been anywhere near prepared enough to respond the way we did as an organisation and me personally as a piece in that jigsaw.* [31]

Taking part in realistic simulation exercises helped individual responders to understand the pressures that the patient-facing teams would have to cope with during a mass casualty response. This highlighted the importance of emergency preparedness training and generated the momentum for change through on-going discussions around needed changes to current practices to improve the system's ability to cope with a large number of casualties, pre-determined by a new casualty distribution plan.

*…For the first time for five years, people got together to discuss …it probably kick started everybody into realising how important this was and what the importance of the need for major incident training and rehearsals.* [33]

*We were slightly worried that that would be a very large number, but the exercise was like, well, actually we can probably cope with this… So it forced, you know, the emergency department, it forced the theatres, it forced intensive care, it forced blood bank and radiology … So it did focus the mind very well so that everyone was actually thinking this is potentially a possibility.* [18]

An opportunity to practise a mass casualty distribution plan was seen by participants as one of the main exercises’ contributions to the response:

*This exercise tested the greater Manchester mass casualty activation plan and this is the exact plan that was executed on the night of the arena bombings. It was vitally useful that night having had that training a few weeks before. [open ended survey response]*

### 4.4 Knowing what to do

Exercise participants reported knowing their roles in the response as well as having confidence in their colleagues’ abilities to perform their roles due to their recent exercise attendance:

*So when I came in that night, there were people there that I'd seen in Operation Socrates and we looked at each other and went, okay, we've done this. Let's get on.* [20]

*We tested the system to receive the patients that we knew we were likely to receive. And therefore we knew how we wanted to manage the trauma teams. We knew how we wanted to use radiology in the assessment of those patients. We knew we wanted to be comprehensive in the use of CT scanning, and we'd work to, you know, to refine the times of transfer for patients to get CTs. We recognised that we needed to improve the radiology input into our major incident plan. And we'd done that in part of the exercise earlier.* [32]

Participants reported that other responders who did not take part in the exercises also benefited from working with their colleagues who took part:

*I think having spoken to some of my colleagues who are also consultants, some of them actively wanted to participate in the exercises and actually responded on the night. And some of my colleagues have been very much, well we're glad you were there because you knew what you were doing.* [18]

Participants specifically highlighted the advantage of having had a recent practise of the response through the operation-based exercise as they could still remember their roles and expected actions rehearsed at the exercise, which was found reassuring.

*I think it was fresh in our heads ….the numbers are very similar to the numbers in the Manchester Arena. So I think when the Manchester Arena thing happened, we'd all been practising it a few weeks before that… So it was fresh in everybody's head. They knew what to do. They knew what their role was.* [22]

However, it was not clear if benefits of recent exercising, when everything was so fresh in peoples’ minds, would have remained if more time had elapsed between the exercise and the response:

*I panicked for the first couple of minutes and didn't really know what to do until I found my action card and I just fell into training mode. I set up the control room, turned all the computers on, put the news on the TV that we have in the control room and phones were all switched on. I just kind of then worked like clockwork. Had that been say that happened six months after the training exercise, would it have been the same? I don't know, that's hard to say.* [31]

### 4.5 A predetermined plan

A new mass casualty distribution plan, that prior to the attack was still in the process of developing and refining, was introduced to coordinate the Manchester Arena bombing response. The plan was designed to improve the care of severely injured patients, by ensuring each patient reaches the right hospital in the appropriate time for optimal care. For this plan to work well, the capabilities of receiving hospitals to deal with a pre-determined number of casualties needs to be known. Both exercises were acknowledged as an opportunity to test the plan, and that experience proved to be essential in developing confidence in this new plan:

*And having made a plan, I think the next crucially important step was that that plan was tested with the help of, you know, the PHE in both Exercise Elsa... But more specifically, Exercise Socrates …Tested the dispersal plan. Delivered patients to individual hospitals, and those individual hospitals then tested their own major incident plan against the case mix of patients that had been designated to them by the mass dispersal plan. And I think the learning from that was critical in, you know, sort of reflecting a more robust response during the actual major incident itself.* [32]

The learning from the exercises that the numbers of patients identified in the mass casualty distribution plan could be effectively managed at the receiving hospitals in the first 2 h of an incident supported the decision to adopt this new plan to guide the healthcare system response in the Manchester Arena incident:

*But because of the feedback from Socrates, because of the understanding that we have a plan that seemed to work, it was decided on the night of the Arena bomb that the plan should be followed. And, you know, and that… That decision proved, I think, to be successful. That decision wouldn't have been taken had we not got confidence in the results of the Exercise Socrates, as to the functionality of the plan.* [32]

The mass casualty distribution plan was used to coordinate the Manchester Arena bombing and was acknowledged as an effective way of managing a large number of severely injured casualties from the scene of the incident. The advantage of knowing the category and number of casualties allocated by the plan was highlighted by hospitals:

*There was no time wasted between... The ambulance control didn't have to phone the hospitals to say can you take ten patients? The hospitals just knew they were going to take ten patients and that was it. So that saved lots of time and bother. It worked great, really.* [22]

*The advantage for us as a Trust was, we knew what type of casualties we should receive, so again, it gave our doctors and nurses a bit of a heads-up.* [19]

The exercise helped many participants to become aware of the major incident plan and what would be expected from their trust and department. Participants emphasised how helpful it was to have a workable plan to guide the response:

*We had an excellent major incident plan that was executed like clockwork. [open ended survey response]*

*Whole process seemed to run as per the plan which was reassuring and helpful. [open ended survey response]*

### 4.6 Real-time modification of response

Learning about health system capabilities as well as limitations in dealing with large number of casualties developed from exercise participation, gave responders the confidence to introduce dynamic changes to existing practises (managing patient flow, imaging, supply of blood, treating children and parents together, patient priority for surgery) on the night of the response, that proved to be effective and saved lives:

*So they actually instigated effectively a one-way flow system which in the night of the Arena is exactly what they did and it worked really well. We cohorted parents with children which no one else would… It had not even been considered beforehand… And certainly we did that, and we've done an exercise subsequent to it where actually children presented to the adult department. And it was, well do you send them to the adults? No. You see them, you treat them, and they go where they need to go.* [18]

*The personal one is leaving space for the head injuries in theatre. That saves lives… I didn't know that beforehand, that changed my decision making on the night. I am very clear that I behaved differently that night in terms of decision making for which patient is going to theatre and I think that made a difference. I know that bit.* [20]

*Again, I'll just point to the transfusion as being a really hot solution that came out of a challenging exercise issue that then paid massive dividends on the night.* [32]

### 4.7 Addressing lessons from exercises

Responders acknowledged the importance of addressing limitations identified from the exercises promptly. The ability to do that prior to the response, even though there had not been much time in between, allowed them to deliver more effective response:

*IE: I think the fact that we challenged the system and ironed out a few very basic, easy to fix niggles meant that we weren't dealing with those challenges on the night, and we could focus on blood provisions to patients, which is what we're actually there for.*

*ES: Yes, very basic, but in fact they ended up to be very effective.*

*IE: Yes.*

*ES: And saved lives.*

*IE: Yes, they did. Yes. Made a big difference. [08]*

## 5 Discussion

The comparative rarity and unpredictability of major incidents makes it difficult to study the contribution of particular training or preparedness activities on the effectiveness of the response which explains reasons behind the lack of empirical evidence on HEPEs impact on a real major incident response. In this study, due to the short time interval between the exercises and the incident (within two months of each other), the direct impact of the exercises on the response became possible to study. To our knowledge, no other major emergency preparedness interventions took place in between. The study applied a strict methodological framework for the data collection and analysis to understand the direct impact of HEPEs on the response to a subsequent mass casualty terrorist incident in the UK. Results of this study provide empirical evidence that HEPEs delivered two months prior to the Manchester Arena bombing had a significant positive impact on the Greater Manchester health system response to this mass casualty incident, and the exercises’ effectiveness were further demonstrated via an educational model [35].

Healthcare staff who took part in the exercises felt significantly better prepared by their emergency preparedness training to respond than those who did not attend the exercises. The study provides further evidence that even for very experienced staff, there are additional benefits from exercise participation: staff learned about plans and put them into practice; they became more able to identify limitations in plans and developed a better understanding of how such limitations could be addressed. A significant number of exercise participants made explicit claims that participation in the exercise made a positive difference to their ability to respond during the subsequent mass casualty incident, with an opportunity to practice the response being one of the most commonly identified enabling factors, particularly for hospital based operational staff. These results highlight the importance of experiential learning in preparing emergency responders [28,29,47], and with infrequent opportunities to take part in a real major incident, emergency preparedness exercises provide an alternative, allowing opportunities to practice how to respond in simulated incidents. The recency of the exercises were highlighted as an important enabling factor, similar to the earlier reports [10,14,48].

Participants’ job experience and support provided by their teams, leadership and more experienced colleagues may account for no difference observed between exercise and non-exercise groups in the survey data with respect to the self-reported clarity of staff roles, team roles and the perceived effectiveness of staff performance of their emergency duties/roles in the Manchester Arena bombing response. Moreover, the study also identified over-reliance on experienced staff who have recently attended HEPEs in this response. The study also provides further evidence of the importance of local major incident plans that supported the response well and were followed. Having clear instructions provided by the plan and action cards offered staff a framework to operate within; this reportedly reduced the level of stress associated with a major incident and supported staff in their actions particularly at the early stages of the response, such as setting up the Incident Command Centre and response teams, clearing the emergency department and calling for extra staff [49].

Major Trauma Centres engage their staff in regular departmental exercises to practice clinical skills and team performance [49]. Such exercises were praised for enabling staff developing knowledge of a mass casualty response and practise their roles in a safe environment with their colleagues. To enhance these local exercises effectiveness such training can include learning of emergency plans, as a significant difference in knowledge of plans was identified between exercise and non-exercise survey respondents in this study.

A variety of specific systemic benefits from exercise participation were reported by health responders to the Manchester Arena bombing. These could be grouped into two major themes of factors that facilitated the response: 1) an *opportunity to practice*: a) a similar simulated incident through the operation-based Exercise Socrates; b) a new mass casualty distribution plan in the recent exercises Elsa and Socrates; and 2) a workable mass casualty distribution *plan* that supported the response well. Learning through practicing a system-wide response to a mass casualty incident in both exercises gave EPRR and clinical staff confidence that the numbers of patients identified in the plan could be managed successfully by the receiving hospitals. That learning contributed to a system-wide change in the response by adopting a new plan that resulted in a more coherent and efficient response in the Manchester Arena bombing [1]; this can be regarded as one of the main system benefits from these exercises. Effective changes to practices, supported by the exercise experience, and introduced on the night of the response, improved the effectiveness and timeliness of the clinical response and ultimately resulted in saving lives. The study provides further evidence to support an earlier made statement that these exercises “were valuable in testing plans and informing policy and practice during the incidents” [14].

Practical experience provided by both exercises produced multiple cognitive learning benefits for staff that facilitated their response, such as self-reported improved knowledge of roles and system-response in a major incident, developed understanding of system limitations that facilitated decision-making and improved knowledge of plans; these benefits are consistent with earlier reported benefits from HEPEs [4]. Other exercise benefits included improved attitude to emergency preparedness training that generated momentum for change before the response, and confidence to introduce changes during the response to the existing practices as well as confidence in multi-team work and system support. These later factors are rather of affective nature, and as reported by participants positive attitudes and improved confidence generated behavioral changes that benefited the response. The data collected in this study confirms that through practical experience in both simulation exercises, discussion-based and operation-based, participants developed learning in both cognitive and affective domains [50,51] that resulted in their improved abilities to respond on the night of the attack. However, whether this learning from exercises remain over time is not known; participants specifically acknowledged the temporal relationship between the exercises and the incident as a very important factor that facilitated the response.

Applying Kirkpatrick's [35] model of evaluation of educational interventions to both exercises, our data shows that the exercises were effective with respect to all four levels of the model. Levels one (*Reactions*) and two (*Learning*) were clearly addressed, as participants reported that the exercises were useful and increased their knowledge and confidence and that they could articulate new concepts they had learned from the exercises (including learning benefits relating to planning, response roles and awareness of partners and major incident response systems). Typically, these levels are the easiest to evaluate when studying complex training interventions. However, the nature of this study meant it was able to show benefits in Kirkpatrick level three outcomes (*Behaviour),* such as changes to practices, some of which were implemented during the response.Further, level four outcomes (*Results)* were also reported, as a new mass casualty distribution plan was introduced, facilitating a more coordinated and timely response. Study participants reported this having positive impacts on the actual response to the Manchester Arena bombing attack.

Single-loop learning post-exercise was clearly observed among individuals in this study, with participants reporting changing aspects of their own practice and aspects of the system within their locus of control during the response, allowing them to deliver more effective response. However, double-loop learning was less clearly visible from the data. Kitson et al. (1998) report successful implementation of learning is a function of the relations between the nature of the evidence, the context in which the proposed change is to be implemented, and the mechanisms by which the change is facilitated [52]. HEPEs affect organisational learning through providing evidence and it can be argued that the context and mechanism are beyond the influence and remit of HEPEs. The main challenge of influencing the application of lessons identified post-exercise may be in the inherent difficulty of manipulating the context of a multi-agency community of practice healthcare emergency response system [53]. The combination of good evidence, in a good context with good facilitation achieve the most successful implementation, but good evidence with good facilitation can overcome a poor context [52]. Observed in this study small-scale practice change and large-scale policy change in the response clearly point out that at least two factors were provided – good evidence from exercises and good facilitation of change. Exercises therefore have the potential to be a driving force for positive system change to improve preparedness, in addition to providing staff with opportunities to practice their response in simulated exercises.

### 5.1 Strengths of the study

To our knowledge this is the first study that reports methodologically collected quantitative and qualitative data on the direct impact of emergency preparedness exercises on healthcare staff response to a large mass casualty incident in the UK. This phenomenon is recognised as difficult to study and the evidence is limited, but the study generated sufficient quantitative data to allow appropriate statistical analysis to be undertaken, and interviews with 21 healthcare staff allowed for confidence that qualitative data saturation had been achieved. Views of health staff across various roles were obtained, and the data reflects the level of heterogeneity typical for this community of practice, as well as the professional diversity found in real incident responses and HEPEs [1,26]. This study complements and extend previous work in this area. Multiple immediate benefits from HEPEs were reported previously [4] but it was not clear how these benefits might translate into a real major incident response. Evidence collected in this study supports the importance of regularly testing a major incident plan in simulated exercises, and particularly in operation-based exercises, to develop both system and staff confidence in plans, as well as to have a workable document to guide the response to a real incident [11]. Importance of providing staff with a regular opportunity to practice responding to a major incident in simulated exercises is supported by the study data; the temporal relationship between the exercises and the incident was identified as an important enabling factor. By applying educational theory, the study demonstrated that learning in both cognitive and affective domains was achieved through experience in recent exercises, which had an impact on individual responders' ability to provide a more effective and coherent response. According to Kirkpatrick's [35] model of evaluation of educational interventions, both exercises, discussion-based Exercise Elsa and operation-based Exercise Socrates, were effective with respect to all four levels of the model. The mixed methods study design allowed more detailed exploration of the phenomenon to be undertaken.

### 5.2 Limitations of the study and further research

Despite the significant contribution of this work, there are some notable limitations to this study. Selection bias [54] may have occurred when collecting data via an on-line survey, and the study relies on self-reported data. Analysis of variance did not find significant difference in the perceived effectiveness of the response between exercise and non-exercise participants, which can potentially be due to the sampling issue. However, multiple benefits from exercise participation were identified from qualitative interviews which are consistent with earlier reported benefits from HEPEs [4] as well as benefits from practicing in emergency preparedness exercises on a response to a real major incident [1,10]. Even though the survey design involved consultations with project stakeholders and included factors that were reported as improved from exercise participation, further work could refine variables to study the exercise's impact on a real major incident response quantitatively for different types of exercises, taking into accounts factors identified in this study. Four-point scale used to collect the survey data may not provide the required sensitivity to identify differences in perceptions of experienced response staff and using an extended scale format would be advocated [25]. Due to the small size of the exercise group, which included participants from both exercise types, the detailed analysis of different exercises' impact on the response was not possible to study. Survey responders were pre-dominantly NHS staff in operational roles, so it was also not possible to explore the participants' characteristics impact on their perceptions. There was little ethnic diversity among study participants, which primarily identified themselves as White British. Also, not all clinical specialities were represented, and most notably absent were junior doctors (those who were in any stage of postgraduate training and had not yet qualified as specialty consultants). Response to the survey from the London incidents was very low, and so this study focused primarily on understanding the impact of exercises on the Manchester Arena bombing response. This study could not clarify the long-term impact of exercises; it is not clear if the impact of the exercises on the response remained the same had there been longer time between the exercises and the response. The optimal frequency and type of health emergency preparedness exercises remain to be determined.

## 6 Conclusion

HEPEs are an important element of emergency preparedness training for healthcare staff. To our knowledge, it is for the first time when the direct impact of a health emergency preparedness exercise on a real mass casualty incident response became possible to measure. The study provides strong objective evidence that the response to the incident was enhanced by the training and service development achieved through emergency preparedness exercises. The main advantage of taking part in HEPEs for healthcare staff appeared to be an opportunity to practice how to respond to a major incident using a major incident plan. Due to the infrequency of mass casualty major incidents, simulation exercises are the only option to test major incident plans, to keep response skills and knowledge updated, and to develop systemic response capabilities. Advantages for individuals to take part in exercises included: understanding individual roles and the roles of teams and department; understanding of the system response and developing confidence in their abilities to respond through practical experience. System level advantages included: testing response plans, identification of system strengths and limitations, and developing clarity about available resources. Provision for regular exercises must be in place to allow healthcare staff at all levels and departments (including junior staff, radiology, pathology, and psychological support and bereavement teams) to regularly practise their response roles with response partners using major incident plans. Doing so will develop understanding and confidence in both individual and system responses to actual events.

## Conflicts of interest

Authors Skryabina, Amlôt and Riley were employed by Public Health England's Emergency Response Department when the emergency preparedness exercises were delivered. However, they were not part of the exercise delivery teams.

## References

[1] Kerslake R. (2018). The Kerslake Report: an Independent Review into the Preparedness for and Emergency Response to, the Manchester Arena Attack on 22nd May 2017. https://www.jesip.org.uk/uploads/media/Documents%20Products/Kerslake_Report_Manchester_Are.pdf.

[2] Torjesen I., Gulland A. (2017). Manchester doctors describe aftermath of bomb blast as NHS continues to treat casualties. BMJ.

[3] NHS England Core Standards for Emergency Preparedness (2018). Resilience and Response. https://www.england.nhs.uk/publication/nhs-england-core-standards-for-emergency-preparedness-resilience-and-response/.

[4] Skryabina E. (2017). What is the value of health emergency preparedness exercises? A scoping review study. Int. J. Disaster Risk Reduct..

[5] Biddinger P.D. (2010). Public Health Emergency Preparedness Exercises: Lessons Learned.

[6] Emery R.J. (2009). Surge capacity volunteer perspectives on a field training exercise specifically designed to emphasize likely roles during a disaster response. Health Phys..

[7] Morris J.G. (2012). Southeastern center for emerging biologic threats tabletop exercise: foodborne toxoplasmosis outbreak on college campuses. Biosecur. Bioterrorism.

[8] (2011). Evaluation of Exercises Handbook.

[9] Fowkes V. (2010). Exercises in emergency preparedness for health professionals in community clinics. J. Community Health.

[10] Hirsch M. (2015). The medical response to multisite terrorist attacks in Paris. Lancet.

[11] Verni C. (2012). A hospital system's response to a hurricane offers lessons, including the need for mandatory interfacility drills. Health Aff..

[12] Sendai Framework for Disaster Risk Reduction 2015-2030, U. Nations, Editor.

[13] (2014). Handbook on Simulation Exercises in EU Public Health Settings.

[14] Moran C.G. (2017). Lessons in planning from mass casualty events in UK. BMJ.

[15] Peterson D.M., Perry R.W. (1999). The impacts of disaster exercises on participants. Disaster Prev. Manag.: Int. J..

[16] Frykberg E., Weireter L., Flint L. (2010). 10 questions and answers about disasters and disaster response. Bull. Am. Coll. Surg..

[17] Klima D.A. (2012). Full-scale regional exercises: closing the gaps in disaster preparedness. J. Trauma Acute Care Surgery.

[18] Biddinger P.D. (2008). Using exercises to identify systems-level preparedness challenges. Publ. Health Rep..

[19] (2017). WHO Simulation Exercise Manual.

[20] Lord E.J., Cieslak T.J. (2004). Lessons learned. Joint regional exercise ("JREX") 2000. Disaster Manag. Response.

[21] Mackenzie C. (2009). How will military/civilian coordination work for reception of mass casualties from overseas?. Prehospital Disaster Med..

[22] High E.H. (2010). Promoting community preparedness: lessons learned from the implementation of a chemical disaster tabletop exercise. Health Promot. Pract..

[23] Macario E. (2007). Preparing public health nurses for pandemic influenza through distance learning. Publ. Health Nurs..

[24] Sarpy S.A. (2005). Simulating public health response to a severe acute respiratory syndrome (SARS) event: a comprehensive and systematic approach to designing, implementing, and evaluating a tabletop exercise. J. Publ. Health Manag. Pract..

[25] Perry R.W. (2004). Disaster exercise outcomes for professional emergency personnel and citizen volunteers. J. Contingencies Crisis Manag..

[26] Savoia E. (2009). Impact of tabletop exercises on participants' knowledge of and confidence in legal authorities for infectious disease emergencies. Disaster Med. Public Health Prep..

[27] Bartley B., Fisher J., Stella J. (2007). Video of a disaster drill is effective in educating registrars on the hospital disaster plan. Emerg. Med. Australasia (EMA).

[28] Caum J., Alles S. (2013). Ready or not: analysis of a no-notice mass vaccination field response in Philadelphia. Biosecur. Bioterrorism Biodefense Strategy, Pract. Sci..

[29] Freimuth V.S. (2008). Action, not talk: a simulation of risk communication during the first hours of a pandemic. Health Promot. Pract..

[30] Coles E. (2014). Learning the lessons from major incidents: a short review of the literature. Emerg. Planning Coll..

[31] Savoia E., Agboola F., Biddinger P.D. (2012). Use of after action reports (AARs) to promote organizational and systems learning in emergency preparedness. Int. J. Environ. Res. Publ. Health.

[32] Drupsteen L., Guldenmund F.W. (2014). What is learning? A review of the safety literature to define learning from incidents, accidents and disasters. J. Contingencies Crisis Manag..

[33] Carroll J.S., Fahlbruch B. (2011). The gift of failure: new approaches to analysing and learning from events and near-misses. Saf. Sci..

[34] Birkland T.A. (2009). Disasters, lessons learned, and fantasy documents. J. Contingencies Crisis Manag..

[35] Kirkpatrick D. (1998). Evaluatinig Training Programs: the Four Levels.

[36] (2016). Emergo Train System.

[37] Johnson R.B., Onwuegbuzie A.J., Turner L.A. (2007). Toward a definition of mixed methods research. J. Mix. Methods Res..

[38] Gulland A. (2017). Leading anaesthetist praises NHS response to Westminster attacks. BMJ.

[39] Craigie R.J. (2018). Manchester Arena Bombing: Lessons Learnt from a Mass Casualty Incident.

[40] Gulland A. (2017). “It wasn't a medical miracle—we made our own luck”: lessons from London and Manchester terror attacks. BMJ.

[41] Creswell J.W., Plano Clark V., Sage L. (2011). Designing and Conducting Mixed Methods Research.

[42] Creswell J.W. (2009). Research Design: Qualitative, Quantitative, and Mixed Methods Approaches.

[43] Jick T. (1979). Mixing qualitative and quantitative methods: triangulation in action. Adm. Sci. Quart..

[44] Bengtsson M. (2016). How to plan and perform a qualitative study using content analysis. Nursingplus Open.

[45] Hsieh H.-F., Shannon S.E. (2005). Three approaches to qualitative content analysis. Qual. Health Res..

[46] Braun V., Clarke V. (2006). Using thematic analysis in psychology. Qual. Res. Psychol..

[47] Silenas R. (2008). Developing disaster preparedness competence: an experiential learning exercise for multiprofessional education. Teach. Learn. Med..

[48] Charles M.T., Settle A.K. (1991). United Flight 232: sioux City's response to an air disaster. Ind. Environ. Q..

[49] Dean E. (2017). When disaster strikes: how emergency servises provide a rapid response. Emerg. Nurse.

[50] Adams N. (2015). Bloom's taxonomy of cognitive learning objectives. J. Med. Library Assoc.: JMLA.

[51] Bloom, B.S., Taxonomy of Educational Objectives: The classification of Educational Goals. 1956, New York: Longmans, Green.

[52] Kitson A., Harvey G., McCormack B. (1998). Enabling the implementation of evidence based practice: a conceptual framework. Qual. Health Care : QHC.

[53] Kothari A. (2015). Communities of practice for supporting health systems change: a missed opportunity. Health Res. Pol. Syst..

[54] Berg N. (2010). Non-response bias. Encycl. Soc. Measur..

