# Supplementary material for: The role of emergency preparedness exercises in the response to a mass casualty terrorist incident: A mixed methods study
Source: Int J Disaster Risk Reduct. 2020 Jun;46:101503. doi: 10.1016/j.ijdrr.2020.101503 (PMC7709486; doi:10.1016/j.ijdrr.2020.101503)
Supplement: Multimedia component 2 [file mmc2.docx]

**Supplementary material**

**File 1**: On-line survey of responders [to the Manchester Arena Bombing]

Background info

1. What is your day job?
2. Where is this job based (CCG, Acute Trust, Primary Care, Local Authority, Ambulance Service, NHS England; other, please specify)
3. For how long have you been in your current role (years)?
4. What is your primary role in a major incident?
5. How many times have you been involved in a major incident response?

In the context of responding to the [name of the incident, e.g. Manchester Arena bombing on the 22 May 2017]

1. At what level was your emergency response role in the Manchester Arena bombing incident? (Operational (Bronze); Tactical (Silver); Strategic (Gold); Other, please specify)
2. What was your primary emergency response role during the Manchester Arena bombing incident?
3. What were your specific duties in your role when responding to the incident?
4. How long were you involved in the response (days)?
5. Are you still involved into the recovery? [Yes/No]
6. Was it clear to you what was expected **from you in your role** in the response? [Yes/No]
7. Was the role of your team clear in this response? Yes/No
8. Was your organisational role clear in this emergency response? Yes/No
9. In your opinion, how effective were you **personally** in performing your emergency duties/role in the Manchester Arena bombing response? (5-point scale)
10. In your opinion, what went really well in this major incident response and why?
11. In your opinion, what went not so well and why?
12. If you were responding to a similar incident in the future what would you do differently and why?

Emergency preparedness training

1. What kind of Emergency Preparedness training have you undertaken over the past three years? In this context, Emergency Preparedness training could include aspects such as major incident roles, specialist response, decision making, log writing and anything else related to emergency planning or business continuity.
2. In the three years prior to this emergency what training that you had attended proved most useful in this response?
3. How well do you think your emergency preparedness training prepared: you personally to respond to this major incident; your team to respond to this major incident; your organization to respond to this major incident; your team to work with other emergency responding teams within your organization; your organisation to work with other responding organisations in this response? (4-point scale)
4. What training did you feel was missing, which if delivered could have improved the response?

Perceived effectiveness in the response

1. Please could you indicate your perceived effectiveness of the following in this major incident response: Your organisation’s Emergency management (Incident Command, Control and Coordination); Your organisation's Internal Emergency communication (department/hospital); Your organisation's External Emergency communication (multi-agency); Dealing with public queries) (4-point scale)
2. Please could you indicate your perceived effectiveness of your organisation's **resource utilisation** in this major incident response: Equipment allocation; Equipment utilization, Equipment effectiveness. (4-point scale)

Perception of resources

1. How well resourced were you in performing your emergency role in this major incident? (4-point scale)
2. How well resourced was your team? (4-point scale)
3. How well resourced was your organisation to respond to the Manchester Arena bombing? (4-point scale)

Impact of emergency preparedness exercises on the response

1. Which of these two emergency preparedness exercises did you attend? [Exercise Elsa; Exercise Socrates]?
2. Did your participation in Exercise Elsa on the 22 March 2017/Exercise Socrates on 29 March 2017 make a difference to your ability to respond to the Manchester Arena bombing incident?
3. How could Exercise Elsa/Exercise Socrates have been improved to help prepare yourself/your team/your organization to respond to a major incident like this?
4. Explain how your participation in Exercise Elsa/Exercise Socrates contributed to your ability to respond to this major incident.
5. What extra useful/relevant skills/knowledge have you learned/developed from taking part in Exercise Elsa/Exercise Socrates which helped you with this emergency response?
6. In relation to your role and that of your team during Exercise Elsa/Exercise Socrates, how cold the exercise be improved for future events?
7. How often do you feel you need to take part in emergency preparedness exercises to improve/maintain your emergency response preparedness: workshop/seminar; simulation discussion-based tabletop exercise; simulation operation-based exercise/drill; full scale field exercise [every 6, 12, 18, 24, 30, 36, no needed]

|  | Emergency response skills   1. What extra useful/relevant skills/knowledge have you learned/developed from taking part in the Manchester Arena bombing incident? 2. What do you think are the most important skills to be efficient in a major incident in your role? 3. Which of these skills can be developed further and how? 4. What key action need to be done to improve the response to a major incident: in your role; within your team; within your organisation? 5. Is there any additional training which would be helpful in improving and maintaining your emergency response skills in the future?   Emergency Plan   1. Does your organisation has a major incident plan? [Yes/No] 2. In your opinion, was this plan useful in supporting this response? [Yes/No] 3. Did you adhere to the plan in this response? [Yes/No] 4. Have you had an opportunity to practise your organisation’s major incident plan over the past three years? [Yes/No] 5. Have you taken part in your organisation's debrief process to identify lessons following the response to the Manchester Arena bombing? [Yes/No] 6. Have any limitations in your organisation’s major incident plan been identified from the Manchester Arena bombing response? [Yes/No] 7. Do you understand how organisational limitations identified in this emergency response will be addressed? [Yes/No] 8. Have you or a representative from your organisation taken part in any multi-agency debrief process following the response to the Manchester Bombing? [Yes/No]   Demographics |
| --- | --- |

1. **What age group are you in?**

18-29

30-39

40-49

50-59

60 or over

Prefer not to say

1. **What is your highest education qualification achieved to date?**

Doctoral degree (e.g. PhD, MBBS etc.)

Masters degree or other post-graduate qualification

Undergraduate degree

A-Levels or equivalent

GCSE’s or equivalent

No qualification

Prefer not to say

Other, please specify

1. **Are you?** Male/Female/Prefer not to say
2. What is your ethnic group?
   1. *English / Welsh / Scottish / Northern Irish / British*
   2. *Irish*
   3. *Gypsy or Irish Traveller*
   4. *Any other White background*
   5. *White and Black Caribbean*
   6. *White and Black African*
   7. *White and Asian*
   8. *Asian / Asian British*
   9. *Indian*
   10. *Pakistani*
   11. *Bangladeshi*
   12. *Chinese*
   13. *Any other Asian background*
   14. *Black / African / Caribbean / Black British*
   15. *African*
   16. *Caribbean*
   17. *Any other Black / African / Caribbean background*
   18. *Arab*
   19. *Prefer not to say*
   20. *Any other ethnic group, please describe*

**File 2:** Semi-structured Interview schedule: The role of exercises in the Manchester Arena bombing response

1. Manchester Arena bombing response experience
2. What was your primary emergency response role in the Manchester Arena Bombing incident?
3. What did this role involve/what were your specific duties?
4. Is this what you had anticipated? Were there any elements you hadn’t anticipated?
5. Did you have a clear understanding of your role? The role of your team?
6. What went well in the actual response and why do you think it went well?
7. What aspects were more challenging to cope with during the response; what about the recovery stage?
8. Do you have any suggestions on how to deal with identified challenges or to better prepare you?
9. Looking back at your experience in the response how well prepared do you think you were in performing your role? How well prepared were your team/organisation?
10. Overall, what experiences have you had to prepare you for managing a major incident; training, exercising, incident response? Day to day job experience. Knowledge of plans.
11. Which of these experiences were most valuable for you in the response; what about preparing you for a recovery stage?
12. **Exercise experience**

Have you taken part in either of the exercises which were delivered in Greater Manchester in March 2017 – Exercise Elsa (22 March 2017) or exercise Socrates (29 March 2017)?

**Yes, to exercise Elsa/[*Socrates]***

- 1. Could you please remind me about Exercise Elsa/[*Socrates]?*

(type, scenario, purpose, your role)

- 1. Can you recall the main issues highlighted during the exercise? Have any of those been resolved in any way? (I can remind them of main points highlighted in exercise report if necessary)
  2. Did any of the issues highlighted in the exercise feature in the Manchester Arena incident response?
  3. Did you learn any useful lessons from Exercise Elsa/[*Socrates]?*personally for you/for your organisation? What kind of lessons? Did you apply any of this learning in the response in any way? Has the exercise had any other sort of impact on your response?
  4. Have you been involved in any post-exercise activities at your organisation to address any of those lessons identified in the exercise prior to the Manchester response (e.g debrief) OR Are you aware of any post-exercise activity within your team/organisation to address any of those lessons? E.g. Have any emergency preparedness plans, relevant to you, changed since the exercise?/

- **If so,** have any of those actions had any impact on the response?

- **If Not**, Was there anything that could have been done post-exercise (as a result of lessons identified) that would have improved your response?
  1. How do you think the lessons identified from the exercise could be most effectively shared and incorporated into practice?

1. **Learning and skills**
   1. Now taking into account your experience with the Manchester Arena Bombing response and looking back at your experience Exercise Elsa /*[Socrates*], was there anything in particular which helped you with your response role/make any difference to your ability to respond? If so, what was that and HOW did it help you?
   2. In your opinion, what are the most important skills and knowledge needed for an effective emergency response in your role and why?
   3. If there were any particular skills that aided your response, how did you develop those skills?
   4. How can these skills be developed/learned in emergency exercises [if not covered]?
   5. What needs to be done to maintain the skills developed in emergency exercise and translate them into a real emergency response?
2. **Retention and transfer**
   1. What was the greatest benefit of Exercise Elsa/ [Socrates] for you/your team?/ How this exercise helped you (your team, your organisation) to be better prepared for the Manchester Arena bombing response?

Was anything missing at the exercise which, if delivered, could have helped you or your organisation in the response or the recovery stage?

- 1. Was there anything else that you, personally, gained from participating in Exercise Elsa/ [Socrates].
  2. Would your response be different if you did not take part in Exercise Socrates?
  3. Would your response to the Manchester Arena incident be different if there were longer time period between Exercise Elsa/ [Socrates] and this major incident? If so, explore in which way.

**4). Perceptions of preparedness**

- 1. Do you feel more prepared to respond to a major incident since participating in the exercise and responding to the Manchester Arena bombing? WHY?
  2. Do you feel prepared to work in a multi-agency context to provide an integrated response to major incidents?
  3. What sort of further training do you think needs to be done to improve a) your personal level of emergency preparedness for a major incident response and recovery, b) your organisation’s emergency preparedness for response and recovery?
  4. How important are emergency exercises in preparing you for your emergency role? Any particular type of exercises? How important are live exercises compared to simulation exercises? If it’s not important, then what is?
  5. How do you think we can improve the educational value of emergency preparedness exercises?
  6. If you were able to choose, when do you think would be a good time to have another exercise and what would that exercise look like?
  7. What advice would you give to someone expected to perform your role in a similar incident?

End of interview.

**File 3:** Semi-structured Interview schedule: The role of exercises in the Manchester Arena bombing response [Ex Elsa or Socrates not attended]

1. **Manchester Arena Bombing response experience**
2. What was your primary emergency response role in the Manchester Arena bombing ?
3. What did this role involve/what were your specific duties?
4. Was that what you had anticipated? Were there any elements you hadn’t anticipated?
5. Did you have a clear understanding of your role? The role of your team?
6. What went well in the actual response and why do you think it went well?
7. What aspects were more challenging to cope with during the response; what about the recovery stage?
8. Do you have any suggestions on how to deal with identified challenges or to better prepare you?
9. Looking back at your experience in the response how well prepared do you think you were in performing your role? How well prepared were your team/organisation?
10. **Exercise experience.**

Have you taken part in either of the exercises which were delivered in Greater Manchester in March 2017 – Exercise Elsa (22 March 2017) or exercise Socrates (29 March 2017)?

***NO to any of these exercises***

Have you been aware about any of these exercises at your organisation?

If **YES:**

- 1. Have you been shared any learning from these exercises? If yes, then in which way?
  2. Have you been involved in any post-exercise activities at your organisation to address any of those lessons identified in the exercise prior to the Manchester Arena bombing response (e.g debrief) OR Are you aware of any post-exercise activity within your team/organisation to address any of those lessons? E.g. Have any emergency preparedness plans, relevant to you, changed since the exercise?

- **If so,** have any of those actions had any impact on the response?

- **If not**, Was there anything that could have been done post-exercise (as a result of lessons identified) that would have improved your response?

- 1. How do you think the lessons identified from the exercise could be most effectively shared and incorporated into practice?

I understand that you did NOT take part in exercise Elsa/Socrates:

1. Have you taken part in any simulation exercises? How useful were they to prepare you for this response (ask separately for discussion-based /operation-based exercises) ? Which elements of those exercises were the most valuable in preparing you for the response?

2. Have you taken part in any live –field exercises previously; how useful were they to prepare you for this response; which elements of that live exercise were the most valuable in preparing you for the response?

3. Overall experience what experiences have you had to prepare you for managing a major incident; training, simulation exercises or live exercises, incident response? Day to day job experience. Knowledge of plans.

1. Which of these experiences were most valuable for you in the response;

b) what about preparing you for a recovery stage?

1. ***Skills***
2. In your opinion, what are the most important skills and knowledge needed for an effective emergency response in your role and why?
3. If there were any particular skills that aided your response, how did you develop those skills?
4. How can these skills be developed/learned in emergency exercises [if not covered]? What kind of exercises will be more appropriate to develop those skills (discussion-based simulations, operation-based simulations, live-exercises)?
   1. What needs to be done to maintain the skills developed in emergency exercise and translate them into a real emergency response?
5. **Perceptions of preparedness**
   1. Do you feel more prepared to respond to a major incident since participating in the Manchester Arena bombing response ? WHY?
   2. Do you feel prepared to work in a multi-agency context to provide an integrated response to major incidents?
   3. What sort of further training do you think needs to be done to improve a) your personal level of emergency preparedness for a major incident response and recovery, b) your organisation’s emergency preparedness for response and recovery?
   4. How important are emergency exercises in preparing you for your emergency role? What kind of exercises would be the most valuable? If it’s not important, then what is?
   5. How do you think we can improve the educational value of emergency preparedness exercises?
   6. If you were able to choose, when do you think would be a good time to have another emergency preparedness exercise and what would that exercise look like?
   7. What advice would you give to someone expected to perform your role in a similar incident?

End of interview.
